# Social Isolation and Cognitive Function: Evidence From Older Chinese Americans

**DOI:** 10.1093/geroni/igaa057.1628

**Published:** 2020-12-16

**Authors:** Dexia Kong, Ying-Yu Chao, Fengyan Tang, XinQi Dong

**Affiliations:** 1 Rutgers University, New Brunswick, New Jersey, United States; 2 University of Pittsburgh, Pittsburgh, Pennsylvania, United States

## Abstract

Chinese older adults are particularly vulnerable to social isolation due to various barriers they face in developing/maintaining social networks (i.e. limited English proficiency and transportation barriers) in the U.S. However, the prevalence of social isolation and its potential health consequences in this rapidly growing minority aging population remain poorly understood. To address this knowledge gap, the current study examines the prevalence of social isolation, and the relationship between social isolation and cognitive function among U.S. Chinese older adults. Data were obtained from the Population-based Study of Chinese Elderly in Chicago collected between 2011 and 2013 (N=3,157). A four-item index (including living alone, not married, lack of confidant, and low participation in social activities) was constructed to assess social isolation (range: 0 to 4, a score of ≥2 was used to identify individuals who were most isolated). Cognitive function was measured by five validated instruments (range: -2.8 to 2.0). Nearly 22% of the sample were socially isolated. Multivariable linear regression analysis showed that social isolation accounted for 44% of variance in global cognitive functioning. Chinese older adults with greater levels of social isolation had poorer overall cognitive function (B= -0.05, SE=0.01, p=0.001). Study findings highlight the importance of addressing social isolation in cognitive aging among older Chinese Americans. Culturally tailored interventions facilitating the development of supportive social networks/ support have the potential to mitigate cognitive decline in this population. Future longitudinal studies need to elucidate potential mechanisms underlying the relationship between social isolation and cognitive function. Practice implications will be discussed.

